# Physicochemical Properties and Microbiome of Vineyard Soils from DOP Ribeiro (NW Spain) Are Influenced by Agricultural Management

**DOI:** 10.3390/microorganisms12030595

**Published:** 2024-03-16

**Authors:** Pilar Blanco, Isaac Rodríguez, Victoria Fernández-Fernández, María Ramil, David Castrillo, Marta Acín-Albiac, Irene Adamo, Clara Fernández-Trujillo, Beatriz García-Jiménez, Alberto Acedo, Noemi Calvo-Portela, Andrea Parente-Sendín, Lara Acemel-Míguez, Flora Alonso-Vega

**Affiliations:** 1 Estación de Viticultura e Enoloxía de Galicia (EVEGA-AGACAL), Ponte San Clodio s/n, 32428 Leiro-Ourense, Spain; david.castrillo.cachon@xunta.gal; 2 Instituto de Investigación en Análisis Químicos y Biológicos (IAQBUS), Universidade de Santiago de Compostela (USC), Constantino Candeira s/n, Campus Sur/Campus Vida, 15782 Santiago de Compostela, Spain; isaac.rodriguez@usc.es (I.R.);; 3 Biome Makers Inc., Davis, CA 95618, USAirene.adamo@biomemakers.com (I.A.); beatriz.garcia@biomemakers.com (B.G.-J.);; 4 Área de Edafología y Química Agrícola, Departamento de Biología Vegetal y Ciencia del Suelo, Facultad de Ciencias, Universidade de Vigo, 32004 Ourense, Spainflorav@uvigo.gal (F.A.-V.); 5 Laboratorio de Evaluación y Tecnología Ambiental, Campus da Auga-Campus de Ourense, Universidade de Vigo, 32004 Ourense, Spain

**Keywords:** bulk soil, vineyard, sustainable agriculture, micronutrients, pesticide residues, metagenomics, bacteria, fungi, microbial networks, microbial terroir

## Abstract

Agricultural management influences the soil ecosystem by affecting its physicochemical properties, residues of pesticides and microbiome. As vineyards grow crops with the highest incidence of pesticides, the aim of this study was to evaluate the impact of conventional and sustainable management systems of vineyards from DOP Ribeiro on the soil’s condition. Samples from soils under three different management systems were collected, and the main soil physicochemical properties were evaluated. A selection of 50 pesticides were investigated by liquid chromatography with tandem mass spectrometry. The bacterial and fungal microbiomes were characterized through amplicon sequencing. The results show that organic agriculture positively influences soil pH and the concentration of some nutrients compared to conventional management. Our microbiome analysis demonstrated that transitioning from conventional to organic management significantly improves several BeCrop^®^ indexes related to key microbial metabolism and soil bio-sustainability. Such a transition does not affect soil alpha diversity, but leads to a higher interconnected microbial network structure. Moreover, differential core genera and species for each management system are observed. In addition, the correlation of the microbiome with geographical distance is evidence of the existence of different *microbial terroirs* within DOP Ribeiro. Indeed, sustainable management leads to higher nutrient availability and enhances soil health in the short term, while lowering pesticide usage.

## 1. Introduction

Microorganisms are a key component of soil ecosystems. They are involved in the nutrient biogeochemical cycle, contributing to soil fertility and promoting the growth and the productivity of crops. In addition, the soil microbiome plays a crucial role in the health of plants. On one hand, soil microorganisms represent an important contribution to plant disease resistance [1]. On the other hand, the soil microbiome may be a reservoir for pathogens. The role of the soil microbiome is particularly relevant in viniculture, being one of the factors included in the concept of terroir. Therefore, together with other biological (rootstock, grapevine variety, soil biodiversity and surrounding plants and animals), physicochemical (soil condition), environmental (climate, topography and landscape) and anthropogenic factors (viticultural practices and winemaking style), the soil microbiome is responsible for the unique, distinctive features of wines from a given region [2,3].

Previous studies on microbial communities in vineyard soils and grapevines have revealed the existence of variations at different spatial scales [4,5,6,7,8,9]. In addition, the grapevine microbiome is related to regional wine characteristics and is known as “microbial terroir” [10,11,12,13]. Hence, vineyard soil microorganisms that are present in grapes and later in musts can influence fermentation processes [14]. The main driver of bacterial and fungal communities in vineyard soils is geographic location, followed by agricultural management practices and the soil’s physicochemical properties [13,15]. In particular, pH is one of the most important parameters affecting the structure of soil communities at a global scale [16]. Accordingly, a strong correlation between the soil C:N ratio and pH within bacterial populations has been reported [5,14]. Furthermore, agricultural management practices alter soil physical and chemical properties like pH and consequently, soil biodiversity [17,18]. In the same vein, a previous study has shown that organic farming increases the amount of organic matter, the potassium content and soil microbial biomass, but has other negative effects on soil quality [19]. Other authors found that organic fertilization significantly raised bacterial and fungal abundance compared to mineral fertilization; however, the microbiome was not related to soil physicochemical properties or climatic factors [20]. In terms of soil quality, Meissner et al. [21] reported that organic and biodynamic management practices enhanced soil fertility but also reduced vegetative growth and grape production. In addition, an evaluation of fungal communities in vineyard soils under conventional and biodynamic management has evidenced differences in the types and abundance of species [22].

Regarding plant health, soil can be a reservoir of many phytopathogenic microorganisms that affect vines. Of these, powdery mildew (*Erysiphe necator*), downy mildew (*Plasmopara viticola*), grey mold (*Botrytis cinerea*) and black rot (*Guignardia bedwelli*) are the most concerning ones, especially under certain climate conditions such as high humidity and moderate temperatures. To avoid their detrimental effects on grape quality, winegrowers need to treat grapevines with different active ingredients including organic fungicides, copper salts and sulfur-based products to prevent and/or to mitigate several diseases/pests [23]. Despite the positive effect of these treatments on the quality and productivity of grapevine crops, the overuse/misuse of pesticides represents a threat to the environment and to human health. In addition, it could lead to pest resistance and economic losses. Within the EU, current policies address an important reduction in the number of authorized pesticides (particularly fungicides and insecticides) and the frequency of treatments. These restrictions apply not only to synthetic organic pesticides, used under conventional management practices, but also to some products tolerated in organic production, as is the case of copper salts, limited to a maximum of 28 kg ha^−1^ over 7 years [24]. Although copper does not promote the development of fungicide-resistant strains, it might cause undesired environmental problems. These issues may occur due to its accumulation in soils, migration to aquatic environments and modulation of soil biota [25]. Restrictions on copper treatments represent a serious drawback to farmers, especially in organic viticulture because synthetic fungicides are not allowed, and Cu-based fungicides do not have a suitable substitute yet [26].

Spain is the country with the largest area of vineyards (937.781 ha) in the world [27] and also with the highest percentage of vineyards under organic production. In Galicia (Northwest Spain), vineyards account for an area of 25.000 ha, distributed among five different protected denomination of origin (DOPs). Despite the excellent organoleptic qualities of wines in this area, less than 0.3% of these vineyards are under certified organic management [28]. The combination of mild temperatures and high humidity makes the vineyards of this entire region susceptible to fungi-driven infections. The vineyards from DOP Ribeiro, the oldest DOP in Galicia, are located in the confluences of valleys shaped by the Miño, Avia and Arnoia rivers. DOP Ribeiro has an intermediate amount of pressure from fungi diseases between the DOP closer to the Atlantic coast (DOP Rías Baixas) and those in the oriental part of Galicia (DOP Ribeira Sacra, Monterrei and Valdeorras). To prevent economic losses, the frequency of treatments applied to vineyards is substantial, and the existence of residues of pesticides in soils has been reported in previous studies [29,30]. Nevertheless, the effect of sustainable agricultural management on the soil’s health, including a characterization of its microbiome, has not yet been assessed in this area. However, a positive correlation between organic farming and yeast diversity in grape musts has already been reported [31,32].

Within this framework, the main aim of this research was the characterization of vineyard soils subjected to different agricultural management systems (conventional, transitional and organic). The impact of these practices was assessed through an analysis of the soils’ physicochemical properties and microbiomes, including the diversity of their bacterial and fungal communities. To our knowledge, this is the first study of vineyard soils in Galicia in which the impacts of agricultural management systems on soil condition, microbiome diversity and functionality are assessed. Our results highlight the contribution of organic management to improving soil physicochemical properties, BeCrop^®^ functionality indexes, microbial diversity and structure.

## 2. Materials and Methods

### 2.1. Site Description and Soil Sampling

**Figure 1:**
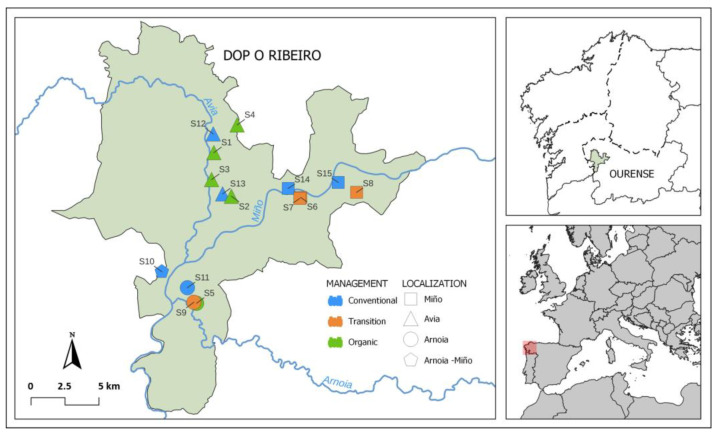
Location of the studied vineyards in DOP Ribeiro (Galicia, NW Spain).

This study was conducted in 15 vineyards located within DOP Ribeiro (NW Spain) area (Figure 1). The annual mean temperature in 2022 was 15 °C, ranging from an average of 5.5 °C in January to 24.3 °C in July (Table S1). The mean temperatures registered for January and February 2023 were 7.2 °C and 6.7 °C, respectively. Regarding precipitation, the area registered almost no rain during summer. On average, August and July registered 8.1 and 6.8 L m^−2^, respectively (Table S2) while autumn months were very rainy (263.2 L m^−2^ in October, 216.4 L m^−2^ in November and 273 L m^−2^ in December). The rainy season continued during winter 2023, registering 206 L m^−2^ in January 2023. The accumulated amounts of rainwater during summer and autumn months in 2022 were 78 and 753 L m^−2^, respectively. An amount of 312 L m^−2^ was recorded since the beginning of 2023 until the sampling date in February 2023 [33].

In order to assess the variability induced by climatic conditions and application of pesticides, soil sampling was carried out in two different seasons (end of July 2022 and middle of February 2023). Considering the management practices in vineyards, the following fields were selected and sampled: 5 vineyards under organic treatment (ORG: S1, S2, S3, S4 and S5), 4 fields in a period of conversion from conventional to organic viticulture (TRA: S6, S7, S8 and S9) and 6 vineyards where conventional practices are applied (CON: S10, S11, S12, S13, S14 and S15). The geographical location of each vineyard was also considered and classified according to nearest river (Arnoia: S5, S9 and S11; Avia: S1, S2, S3, S4, S12 and S13; Miño: S6, S7, S8, S14 and S15). The samples from S10 were considered Arnoia–Miño as both rivers are near this vineyard (Figure 1).

Surface (0–5 cm) and subsurface (5–20 cm) representative soil samples were collected from each vineyard using sterile bags. After removing leaves and grass, 10 randomly distributed subsamples of each depth were collected along the rows of vines and 30–40 cm far from the vine plant. Each subsample location was registered for successive campaigns. The subsamples of each category were merged and mixed in the field to obtain a representative composite sample of each depth (0–5 and 5–20 cm) and vineyard. Once properly homogenized, composite samples were divided into two parts; the smallest one was immediately stockpiled in sterile tubes, maintained at 4 °C in a portable cooler, and sent for molecular analysis to the Biome Makers laboratory in Valladolid (Spain). The remaining soil was sieved and the fraction below 2 mm used for analysis. Sieving was either performed on site (in the summer campaign) or after allowing samples to dry at room temperature for several days to remove the excess moisture. Sieved composite samples were split into two parts to conduct physicochemical analysis and pesticide residue characterization. Microbiome analysis also included soil samples from a preliminary screening in November 2021.

### 2.2. Physicochemical Characterization of Soils

The pH of soils was measured in both distilled water (pHw) and 0.1 M KCl (pHk) soil suspensions with a 1:2.5 soil:solution ratio after equilibration time of 10 min and 120 min, respectively. Total C and N contents were measured in finely soil-milled samples in a Thermo Finnigan 1112 Series NC autoanalyzer.

Exchangeable major basic cations (Na_ex_, K_ex_, Mg_ex_ and Ca_ex_) were extracted from soil samples with 1 M NH_4_Cl solution following Peech et al. [34] while exchangeable acid cation (Al_ex_) was released with 1 M KCl [35]. The concentration in the extracts of Al, Mg and Ca was measured through atomic absorption spectrometry and those of Na and K by atomic emission spectrometry. Based on the charge of the cations (cmol_c_ kg^−1^), the effective cation exchange capacity (CICe = Na^+^ + K^+^ + Mg^2+^ + Ca^2+^ + Al^3+^) and the sum of the concentration of basic cations (SB) were also calculated. Available phosphorous was determined according to Bray and Kurtz [36]. Sand, silt and clay content in mineral fraction of selected samples was determined after applying the pipette method [37].

Aluminum and Fe content distribution was determined according to the method of García-Rodeja et al. [38]. In brief, total free Al and total free Fe (Al_n_ and Fe_d_) were evaluated after being extracted with 0.5 M NaOH and Na-dithionite citrate solutions, respectively. Noncrystalline Al and Fe compounds (Al_o_ and Fe_o_) were extracted with 0.2 M ammonium oxalate–oxalic acid solution, and Al and Fe were complexed using soil organic matter (Al_p_ and Fe_p_) with 0.1 M Na-pyrophosphate solution. The concentration of Al and Fe in each of above extracts was determined through atomic absorption spectrometry.

Total soil Cu_T_, Zn_T_, Mn_T_ and Fe_T_ contents were extracted from milled soil samples through acid digestion (HNO_3_, HF and HCl) in a microwave oven, and the potential availability (Cu_ed_, Zn_ed_, Mn_ed_ and Fe_ed_) was evaluated using EDTA methodology [39] The concentration in the extracts was determined by atomic emission spectrometry. The distribution of Cu and Zn contents was also evaluated after being released with ammonium acetate solution (Cu_a_ and Zn_a_), sodium pyrophosphate solution (Cu_p_ and Zn_p_), oxalic acid–ammonium oxalate solution (Cu_o_ and Zn_o_) and oxalic acid–ammonium oxalate–ascorbic acid solution (Cu_oa_ and Zn_oa_). Through this procedure, the metal distribution among soils fractions can be deduced [40,41]. Exchangeable metal is that directly derived from Cu_a_ and Zn_a_ extractions, and organically bound metal results from subtracting Cu_p_–Cu_a_ and Zn_p_–Zn_a_. The metal bound to amorphous inorganic materials is derived from the difference between Cu_o_–Cu_p_ and Zn_o_–Zn_p_; the concentration bound to crystalline iron and aluminum hydrous oxides is derived by deducting Cu_oa_–Cu_o_ and Zn_oa_–Zn_o_; and finally, residual fraction is derived from Cu_T_–Cu_oa_ and from Zn_T_–Zn_oa_.

### 2.3. Determination of Pesticide Residues in Soil Samples

#### 2.3.1. Sample Preparation

The extraction of organic pesticides (fungicides and insecticides) from sieved soils was carried out using a pressurized-liquid extraction (PLE) device, adapting conditions reported in a previous study [30]. In brief, 2 g of accurately weighed soil was spiked with a selection of isotopically labelled compounds (SSs) (62.5 ng g^−1^), allowed to stand for 30 min, and transferred to 11 mL volume of PLE cells containing 2 g of diatomaceous earth. The remaining free volume was filled with the same material. Extractions were carried out at 80 °C, in a single static cycle, using a mixture of methanol:acetonitrile (MeOH:ACN, 70:30), with cells pressurized at 1500 psi. The primary extract was concentrated and adjusted to a final volume of 5 mL using the same mixture of MeOH:ACN. Extracts were filtered (syringe filters with pore size of 0.22 µm were employed) and stored at 4 °C until analysis. Procedural blanks were prepared by replacing vineyard soils with diatomaceous earth, which was spiked with the selection of SSs, extracted and concentrated under the same conditions as those of the vineyard soils.

#### 2.3.2. Determination Conditions

Determination of pesticide residues in soil extracts was carried out by liquid chromatography (LC) in combination with tandem mass spectrometry (MS/MS). The employed system consisted of a Waters Acquity ultra-performance LC instrument connected with an XEVO TQD from the same supplier, furnished with an electrospray ionization (ESI) source. Compounds were separated with a Zorbax Eclipse Plus C18 type column (50 mm × 2.1 mm, 1.8 µm) purchased from Agilent. Ultrapure water (phase A) and acetonitrile (phase B), both 0.1% in formic acid, were employed as mobile phases at a flowrate of 0.4 mL min^−1^. The temperature of the column was maintained at 40 °C. The composition of the mobile phase was programmed as follows: 2% B (0 min), 50% B (1.3–2.8 min), 100% B (6.4–7.5 min) and 2% B (7.6–10 min). The retention times, together with m:z ratios for precursor, as well as quantification (Q1) and qualification (Q2) ions of each compound, including SSs, are provided in Supplementary Information, Table S3.

Quantification of pesticide residues existing in vineyard soils was performed using solvent-based standards, prepared in the range of concentrations from 1 ng mL^−1^ to 200 ng mL^−1^, with SSs at 25 ng mL^−1^. Identification of compounds in soil extracts was based on retention times and Q2/Q1 ratios matching those obtained for calibration standards within maximum variations of 0.1 min and ±30%, respectively. Concentrations in soil extracts were multiplied by a factor of 2.5 (the ratio between the volume of extract and the mass of soil) and expressed as ng g^−1^, referring to freeze-dried soil. In case of compounds showing E/Z isomers (i.e., the fungicide dimethomorph), the sum of concentrations for both isomers is provided.

#### 2.3.3. Quality Control and Quality Assurance

Quality control experiments involved checking the stability of calibration curves by injection of a 25 ng mL^−1^ standard every 10 injections, analysis of a procedural blank every 10 soils samples, and verification of the accuracy of the procedure with spiked soil samples. Table S4 summarizes the accuracy of the procedure for compounds considered in the analytical procedure.

### 2.4. Soil Microbiome Analysis

#### 2.4.1. DNA Extraction and Library Preparation

After collection, samples were immediately sent for molecular analysis to the Biome Makers laboratory in Valladolid, Spain. DNA extraction was performed with the DNeasy PowerLyzer PowerSoil kit from Qiagen (Hilden, Germany) for the BeCrop^®^ platform [42]. To characterize both bacterial and fungal microbial communities associated with bulk soils, BeCrop^®^ custom primers were used for PCR amplification, specifically targeting the 16S rRNA V4 region and the ITS1 region. Amplicons were purified using the KAPA Pure Beads (Roche, Basel, Switzerland) kit, while correct 16S and ITS amplification was assessed using agarose gel. Purified PCR products were then subjected to library preparation, following a two-step PCR Illumina protocol [43,44]. Next, DNA was quantified using a Qubit fluorometer with Qubit HS Assay Kit 500 (Thermo Fisher Scientific, Waltham, MA, USA). Finally, libraries were sequenced on an Illumina MiSeq instrument (Illumina, San Diego, CA, USA) using 2 × 251 paired-end reads.

#### 2.4.2. Bioinformatic Processing

Primers were removed from paired-end reads using Cutadapt [45]. Then the trimmed reads were merged with a minimum overlap of 100 nucleotides. Next, the sequences were quality filtered using the expected error with a maximum value of 1.0 [46]. After quality pre-processing, reads with single-nucleotide differences were iteratively clustered together to form ASVs (amplicon sequencing variants) using Swarm [47]. De novo chimeras and remaining singletons were subsequently removed [48]. Finally, taxonomy was assigned from ASVs using global alignment with 97% identity against a curated reference database from SILVA 138.1 for 16S sequences and UNITE 8.3 for ITS sequences [49,50].

#### 2.4.3. Computation of Microbiome Indexes and Network Properties

Local network properties were determined following the procedure described by Ortiz-Alvarez et al. [51] Briefly, microbial community networks were built for 16S and ITS samples independently following the methodology reported in a previous publication [52]. Presence–absence meta-network with all samples was built using rarefied counts and the ASV pairs, which occurred significantly more or less frequently than expected, were preserved and determined co-occurrence or co-exclusion network, respectively. Local network properties for both markers were computed for both co-occurrence and co-exclusion: modularity, transitivity and average path length. Modularity outlines the separation among groups of microorganisms (modules) that tend to frequently co-occur or co-exclude in specific ecological niches. Next, transitivity (clustering coefficient) measures the tendency for connected nodes to form closed triangles. Finally, average path length quantifies the degree of connectivity to go from one side of the network to another.

BeCrop^®^ indexes are patented indicators to assess health status of soils based on metagenomics data as described by [53]. Briefly, these indicators assess relevant traits related to soil health ranging from metabolic potential to biocontrol and hormone estimations. BeCrop indexes have been included in previous soil microbiome studies, highlighting the higher quality of the BeCrop^®^ technology [54]. Detailed descriptions of BeCrop^®^ indexes are found in Supplementary Material, Table S5. Widely used Shannon and Chao1 alpha diversity indexes were also included in the current analysis.

### 2.5. Statistical Analyses

Soil physicochemical variability was evaluated according to three parameters: sampling time, depth and vineyard management practices. In addition to descriptive statistics, the influence of the different factors on soil properties’ variability was evaluated through the non-parametric test Kruskal–Wallis (H). The Mann–Whitney (U) test was also applied for pair-wise management comparison. Both tests were conducted using the IBM SPSS Statistics software for Windows (version 25) and *p* < 0.05 was considered for statistical significance.

Microbiome analyses were mainly performed using phyloseq and vegan packages in R [55,56]. Alpha diversity was calculated through Shannon and Chao1 index on rarefied data. Mixed models were fitted to a parametric model containing the main effects of management, time and depth for each microbiome index, network property and physicochemical property, as responses. Ribeiro DO zone was included as a random effect. Models that led to singularity or had high levels of residual autocorrelation were excluded. Marginal (random and fixed terms) and conditional (fixed terms only) R-squared for each model was determined. Next, ANOVA on fixed terms was conducted, and subsequent post-hoc comparison across significant factor levels was performed. *p*-value was corrected for multiple tests using the FDR procedure.

Beta diversity was assessed through principal coordinate analysis (PCoA) using the Bray–Curtis distance. Computed microbiome samples distances were correlated to geographical distances using Spearman correlation. Explained variance of resulting ordination by management, time, depth and DOP Ribeiro zone was determined through PERMANOVA. Prevalence of conserved prokaryotic and fungal genera within soil microbiomes in different management systems was visualized as heatmaps at varying detection thresholds. Shared and exclusive taxa numbers at genus level across management systems are represented in Venn diagrams for both 16S and ITS. Last, differentially abundant (DA) taxa within different management practices were determined on rarefied counts through negative binomial regression at various taxonomic levels with DEseq2 R package [57].

## 3. Results and Discussion

In this study, vineyard soils from DOP Ribeiro under organic, conversion (from conventional to organic) and conventional management systems were compared to assess how these practices influence the soil physicochemical properties, including the presence of pesticides residues and the microbial community. Regarding the microbial community, the microbiome structure was evaluated in terms of the network properties, the BeCrop^®^ indexes and microbial diversity.

### 3.1. Influence of Vineyard Management on Soil Physicochemical Properties

Soil physicochemical properties are relevant factors influencing environmental conditions for soil microbial community structure and assemblage dynamics. Moreover, the sampling time, depth and soil management practices are known to influence the variability in soil conditions, like soil pH, organic matter content, and fertility, among other soil parameters.

In this research, for most of the evaluated soil properties, no significant differences were detected between the sampling dates, as also reported in other studies [58]. Therefore, the data from both sampling campaigns were treated as replicates. Nevertheless, when soil depth was introduced as a factor, the non-parametric Mann–Whitney U test identified significant differences among important soil properties, like the C and N contents, basic exchangeable cations or micronutrient concentrations (Zn_T_, Zn_ed_, Mn_ed_ and Cu_ed_). Our particle size distribution analysis indicated that the studied soils are sandy loam soils, a common texture of soils developed over granitic materials that favor optimal soil drainage.

Table 1 summarizes the descriptive values of pHw, pHk, C, N, C/N and P_bray_ for each soil depth evaluated (0–5 cm and 5–20 cm) and management system for the vineyards.

The soils studied are considered to be moderately acidic, with an average pHw value of 5.76 ± 0.5, ranging from 4.84 at 5–20 cm in a conventional vineyard to 6.91 at 0–5 cm in an organic one. Most of the soils reached a pH of 5.0, below which different macro- and micronutrients’ availability is reduced [59]. Although no differences among the soil depths were detected, if the management system is included as a factor, the pH_w_ at both 0–5 cm and 5–20 cm under organic management is statistically higher than that of the conventionally managed fields (Table 1). The vineyards that are transitioning show intermediate values. The pHk values are slightly lower (average: 5.00 ± 0.41) and range from 4.02 to 5.65. The pHk of the deepest soil depth under conventional practices (4.7 ± 0.3, Table 1) is the lowest. Soil management practices may influence the soil’s reaction and therefore determine the availability of macro- and micronutrients.

The total C contents are always higher (U = 127.00, Sig. < 0.001) in 0–5 cm samples (23.8 g kg^−1^) than in 5–20 cm samples (13.5 g kg^−1^) but no differences were identified among management practices. Similar results were found for the N contents, ranging from 1.03 g kg^−1^ at 5–20 cm to 1.9 g kg^−1^ at 0–5 cm. The total C and N values agree with those already indicated by Fernández-Calviño et al. [40] in vineyard soils from the same region. The ratio of C/N as well as the available P contents (P_bray_) did not differ among the soil sample depths and management practices. Respectively, the average values are 13.1 and 43.3 mg P kg^−1^. However, other studies have reported that vineyards that were under biodynamic or organic management typically had lower C and N contents than those under conventional management [17].

**Figure 2:**
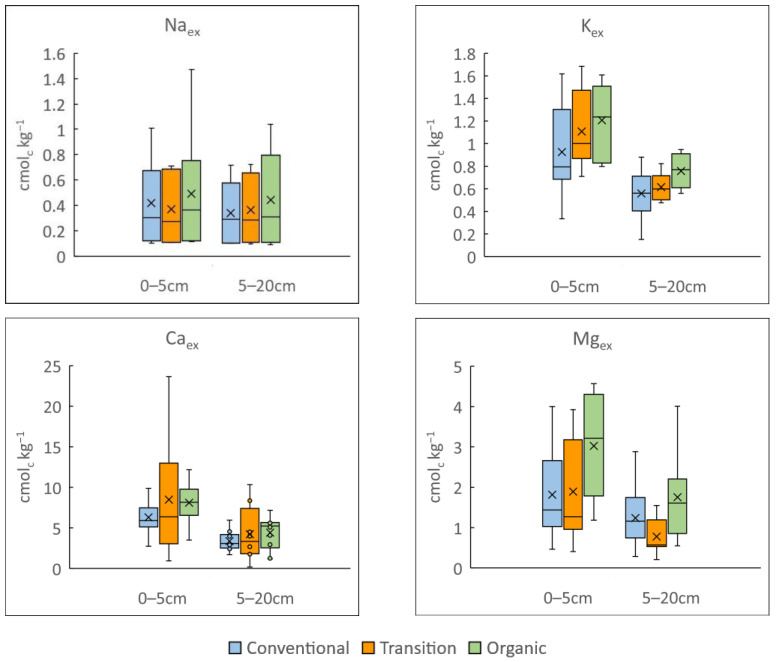
Box and whisker plot of different basic exchangeable cations’ concentration at 0–5 and 5–20 cm soil depths from vineyards under conventional (blue), transitional (orange) and organic (green) management practices. The bottom and the top of the box, respectively, show quartiles 1 and 3 of data set. The line in the box shows the median and the X shows the average of data set. The maximum and minimum are shown with whiskers above and below the box, respectively.

The variability in results derived after the exchangeable base cations’ release is shown in Figure 2. At the 0–5 cm depth, the most abundant exchangeable basic cation is Ca_ex_ (7.5 cmol_c_ kg^−1^) followed by Mg_ex_ (2.2 cmol_c_ kg^−1^), K_ex_ (1.1 cmol_c_ kg^−1^) and Na_ex_ (0.4 cmol_c_ kg^−1^). The same trend, although with lower values, is found for the 5–20 cm depth: Ca_ex_ (3.9 cmol_c_ kg^−1^) > Mg_ex_ (1.3 cmol_c_ kg^−1^) > K_ex_ (0.6 cmol_c_ kg^−1^) > Na_ex_ (0.4 ± cmol_c_ kg^−1^). The values are slightly higher than indicated by Fernández-Calviño et al. [40], especially those from the 0–5 cm depth. The Mann–Whitney U test highlighted the influence of soil depth for the Ca_ex_, Mg_ex_ and K_ex_ concentrations but not for Na_ex_. Following these results, the SB at 0–5 cm (11.2 ± 5.4 cmol_c_ kg^−1^) is higher than that at the 5–20 cm depth (6.2 ± 2.8 cmol_c_ kg^−1^).

When management practices are included as a factor in the statistical analysis, there is evidence of significant differences among soil exchangeable cation concentrations. For both soil depths evaluated, the transitional vineyards showed intermediate values; therefore, a peer assessment without a parametric Mann–Whitney U test but including management practice as a factor, was performed for each exchangeable cation and depth.

When comparing vineyards under organic management with conventional ones, no differences among Na_ex_ concentrations were found, as shown in Figure 2. But at the 0–5 cm depth, exchangeable concentrations of Ca and Mg are higher in vineyards under organic management (green) than in conventional (blue) ones (U = 29.0, Sig.: 0.041 and U = 24.0, Sig.: 0.018; respectively) and are, therefore, also higher than those indicated by Fernández-Calviño et al. [40] in the same region. The SB for the 0–5 cm depth of the organic soils (12.8 cmol_c_ kg^−1^) is also higher than that in the conventional ones (9.4 cmol_c_ kg^−1^). For the 5–20 cm soil depth, the K_ex_ values in the organic vineyard soils are slightly higher than those in the conventionally managed ones.

The Mg_ex_ from the transitional fields at 0–5 cm is similar to that from the conventional ones and lower than that from the organic soils. At the 5–20 cm depth for the transitional soils, the K and Mg values are also lower than those in the organic vineyards and similar to those from the conventional vineyards.

When focusing on elements that are more influenced by the parent material during weathering, like Fe and Al and its fractionation, both soil depth and management practices did not play as an important role as the exchangeable cation content (Figure S1). The average values of the total free Al and Fe (Al_n_ and Fe_d_) were 2.5 ± 1.3 g kg^−1^ and 9.9 ± 7.8 g kg^−1^, respectively. Non-crystalline Al and Fe compounds (Al_o_ and Fe_o_) accounted for 1.6 ± 0.6 g kg^−1^ and 1.3 ± 0.4 g kg^−1^, respectively. Those extracted with 0.1 M Na-pyrophosphate solution are assumed to be complexed by soil organic matter (Al_p_ and Fe_p_) and the respective values are similar among the soil depths and under the different management practices. These results suggest that the differences detected among the properties of soils under the different management practices are not due to influence of the parent material. In addition, the values are within the corresponding ranges established by Fernández-Calviño et al. [40], who evaluated 25 soil samples at a depth of 0–20 cm from vineyards in the same region.

The total contents of relevant micronutrients (Fe, Mn, Cu and Zn) are summarized according to soil depth and management practice in Table 2. Only Zn_T_ shows differences among the soil depths when taking all the management practices into account; those from the 0–5 cm depth (93.7 mg kg^−1^) are higher than those from the 5–20 cm depth (72.2 mg kg^−1^). The concentration at both depths is slightly lower than in that in nearby vineyard soils [60]. In both cases, the values are higher than those shown by Amorós et al. [61] for the Castilla la Mancha region (Spain) but lower than those for the Penedés area (Spain) after an application of manure [62] and those for surface and subsurface soils in Marne, France [63]. Zn-based fungicides [64,65], foliar fertilizers [66] and the application of cattle and pig manure [62] could explain the higher Zn concentrations in the surface soil samples. In all cases, the values in the current study are lower than the generic levels of the studied region [67] and less than 500 mg kg^−1^, the threshold for toxic effects [68].

After a peer assessment considering management practices, for the 0–5 cm depth, the total level of Zn in the organic fields is higher than that in the conventional vineyards (Table 2), as also evidenced in some vineyard fields by Likar et al. [66]. Ferreira et al. [69] indicated that under sustainable management, soils are prone to accumulate nutrients, especially Zn and Cu, at surface depths because of applied fertilizers and restricted tillage practices, which could also explain why the total manganese contents in organic soils are also different than those from the other soils evaluated.

The total Cu contents range from 24.5 to 571.4 mg kg^−1^, but no differences among soil depths and soil management practices were identified. The Cu_T_ average content was 170.5 mg kg^−1^ lower than 227 ± 65 mg kg^−1^ as found by Chopin et al. [63] but within the range indicated by Fernández-Calviño et al. [70] in the same region and DOP.

**Figure 3:**
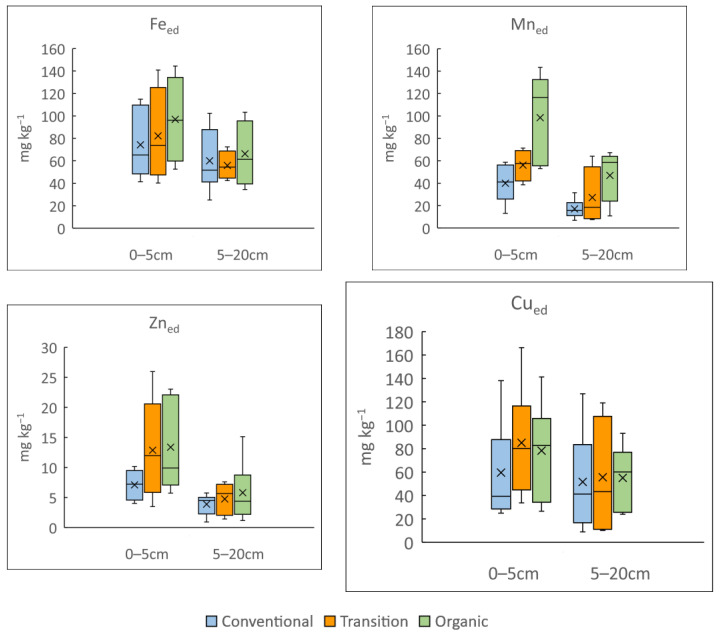
Box and whisker plot of available concentration (EDTA extractions) of Fe, Mn, Zn and Cu in 0–5 and 5–20 cm soil depths from vineyards under conventional (blue), transitional (orange) and organic (green) management practices. The bottom and the top of the box show quartiles 1 and 3 of data set, respectively. The line in the box shows the median and the X shows the average of data set. The maximum and minimum are shown with whiskers above and below the box, respectively.

As the total content is not always a good indicator of the available content, EDTA extractions for determining availability [39] were performed. The results of the variability for Fe_ed_, Mn_ed_, Zn_ed_ and Cu_ed_ are shown in Figure 3. The available Fe and Mn contents are in the same order of those indicated by Brataševec et al. [71], but those of Zn and especially Cu are much higher in this study.

Except for Fe_ed_, the non-parametric Mann–Whitney U test identified differences among the soil depths (Figure 3) for the available contents of Mn_ed_, Zn_ed_ and Cu_ed_. In addition, the Mn_ed_ content in the organically managed fields at a soil depth of 0–5 cm is significantly higher than that in the other vineyards.

**Figure 4:**
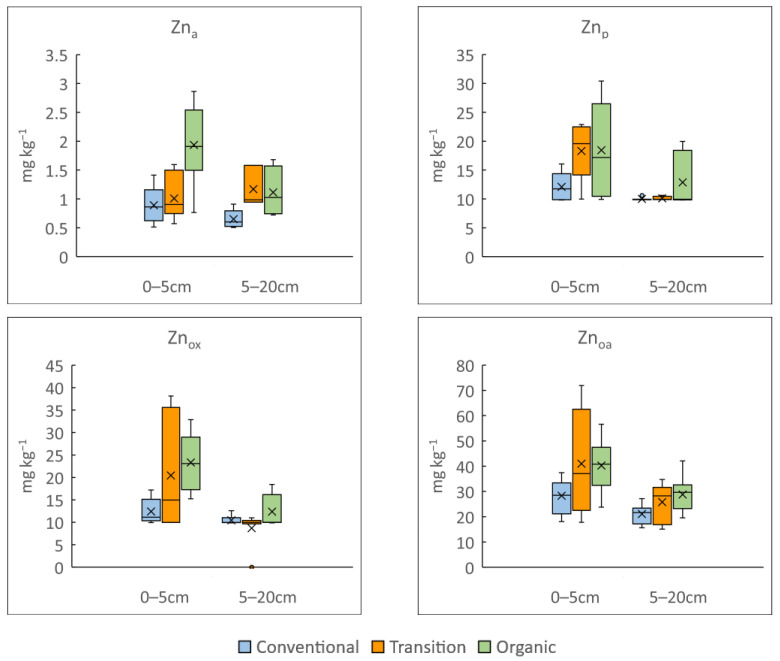
Box and whisker plot of released concentrations of Zn for each depth and management practice after fractionation study with ammonium acetate solution (Zn_a_), sodium pyrophosphate solution (Zn_p_), oxalic acid—ammonium oxalate solution (Zn_o_) and oxalic acid—ammonium oxalate—ascorbic acid solution (Zn_oa_) for 0–5 and 5–20 cm soil depths from vineyards under conventional (blue), transitional (orange) and organic (green) management practices. The bottom and the top of the box show quartiles 1 and 3 of data set, respectively. The line in the box shows the median and the X shows the average of data set. The maximum and minimum are shown with whiskers above and below the box, respectively, when there are no outliers (empty circles).

Due to the differences between the Zn_T_ and Zn_ed_ concentrations in soils under different management practices and because of the importance of Cu as fungicide in vineyard cultures, the fractionation of Zn and Cu was evaluated to better understand the distribution of the total content among the soil fractions. Figure 4 shows the variability in the Zn concentrations for each depth and management practice after the release with the ammonium acetate solution (Zn_a_), sodium pyrophosphate solution (Zn_p_), oxalic acid–ammonium oxalate solution (Zn_o_) and oxalic acid–ammonium oxalate-ascorbic acid solution (Zn_oa_). That of Cu is shown in Figure S2.

Soil depth is not a determining factor for Zn_a_ content, but because of low concentrations of exchangeable Zn, our results must be carefully interpreted. For the sodium pyrophosphate and oxalic acid–ammonium oxalate extractions, both soil depth and soil management did not influence the Cu results (Figure S3). The opposite is true for the Zn results (Figure 4), which indicated soil depth is an influencing factor for Zn_p_ and Zn_ox_. In addition, Zn_ox_ at the 0–5 cm depth for the organic fields is higher than that from the conventional ones.

When the oxalic acid–ammonium oxalate–ascorbic acid extraction was applied, the concentrations of both Cu and Zn were the highest among the fractionation solutions (Figure 4 and Figure S3). Once again, no differences between the Cu released concentrations were detected among soil depths and soil treatments, but for Zn_oa_, the concentration at the 0–5 cm depth was higher than that at the 5–20 cm depth. In addition, Zn_oa_ is higher in the organically managed fields than in the conventional ones at both of the depths evaluated. The surface soils from the organic fields show slightly higher concentrations of Zn_T_, Zn_a_, Zn_p_, Zn_ox_ and Z_oa_ than those from the conventional fields. Nevertheless, the less mobile soil fractions (residual and bound to crystalline Fe and Al hydrous oxides) from any evaluated field management system are those with the highest proportion of Zn (comprising 80%), followed by the organic fraction (15%) (Figure S3). In contrast, organically bound Cu is the highest Cu fraction accounting for around 40% of the Cu_T_ (Figure S3) in every kind of managed field.

For most of the soil properties evaluated and considering the differences between the soil depths and management practices applied, organic management positively influenced the soil properties as trends of decreasing soil acidity are clearly shown. In addition, the values of exchangeable basic cations like Ca and Mg and of micronutrients like Zn are higher in the organically managed fields than in the conventional ones. Other studies [72] have reported a higher concentration of Cu in the soil of an organically managed vineyard, while conventionally managed soil presented a higher concentration of Na and Mg and higher pH values. However, further studies increasing both the number of fields under different management practices as well as the application time for organic management are needed to confirm these observed trends. In this sense, in a long-term study about organic viticulture and soil quality, Coll et al. [19] reported only significant differences after 11 years of organic farming for total organic carbon, nitrogen, available potassium and soil microbial biomass.

### 3.2. Presence of Pesticide Residues in Vineyard Soil from DOP Ribeiro

The residues of pesticides in vineyard soils depended on the cultivar management conditions and sampling dates. In the organic vineyards, residues were noted only in the sampling campaign carried out in the summer of 2022, with the maximum total concentrations below 33 ng g^−1^. The fungicides detected in these samples (codes S1 to S5) were metalaxyl, dimethomorph and carbendazim. Although aerial transport or drift during the application of these compounds in nearby areas might explain their occurrence in the top soil (0–5 cm) of organic vineyards, these species were also found at similar levels in the lower soil layer from 5 cm to 20 cm. During the winter campaign (February 2023), the total residues of the investigated pesticides stayed below 5 ng g^−1^ in all the organic soils.

The concentrations measured in the samples obtained from the upper (0–5 cm) and lower (5–20 cm) layers from the transitional and conventionally managed vineyards for the two sampling campaigns are provided as Supplementary Information (Table S6). Out of the 50 compounds investigated in this research, 25 pesticides were found above their limits of quantification [30] in at least one of the samples. With the exceptions of acetamiprid and methoxyfenozide, the rest of the compounds are either employed as fungicides or they are known transformation products of fungicides, as it is the case of carbendazim or CGA 62,826, generated from the environmental transformation of methyl thiophanate and metalaxyl, respectively.

**Figure 5:**
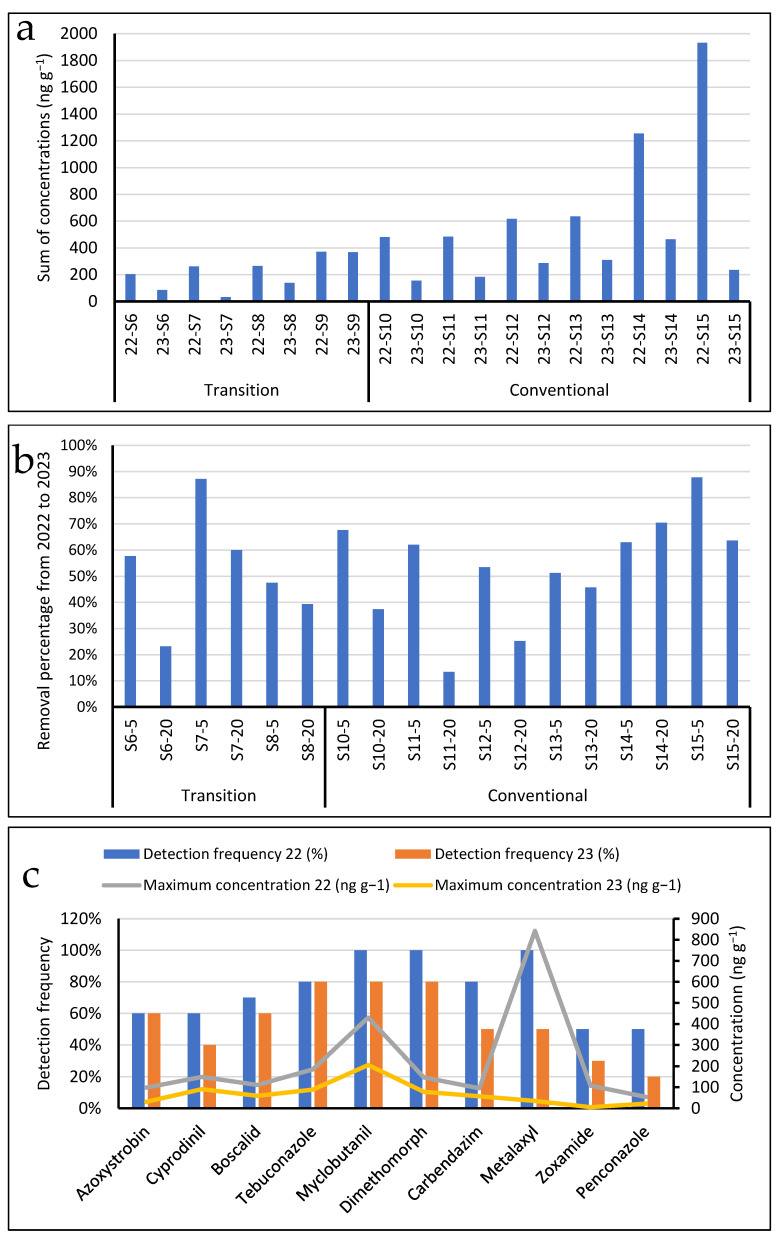
(**a**) Sum of pesticide residues in the top layer of transitional and conventionally managed vineyards; (**b**) Reduction in pesticide residues in top (0–5 cm) and lower layers (5–20 cm) of soils from July 2022 to February 2023; (**c**) Detection frequencies and maximum concentrations of selected compounds in the set of processed vineyard soils.

Regarding the total pesticide residues, the differences between the soils obtained from cultivars labelled as transitional and conventional were not as evident as for the samples from the organic vineyards. For instance, considering the samples taken in the campaign from July 2022, the concentrations in vineyard S9 (code 22-S9) were quite similar to those found in plots S10 and S11 (codes 22-S10 and 22-S11) (Figure 5a). In fact, when comparing the residues measured the following winter, S9 (the transitional management vineyards) was the only place where the pesticides levels were not attenuated. The mechanical movement of the soil between sampling campaigns or with a later treatment with antibotrytic fungides at the end of the summer after the first sampling campaign might be responsible for the anomalous data obtained for the upper soil layer at sampling point S9 in the two campaigns.

Figure 5b summarizes the reduction in the concentrations of pesticides measured in the top (0–5 cm) and bottom (5–20 cm) layers of the transitional and conventional vineyards, except those at sampling point S9 from summer 2022 to winter 2023. As a general trend, the reduction in the total pesticide content was lower in the layer from 5 to 20 cm than that in the top layer (0–5 cm). This pattern might be explained considering the migration of compounds from the upper to the lower layer. Other variables contributing to this differential dissipation could be the changes in the microbiological activity of soil as function of depth, the different penetration rates of pesticides in soil depending on its texture, the slope of each vineyard and the particular properties of each compound, including their soil mobility and/or environmental persistence.

The detection frequencies and maximum concentrations for the compounds showing values above 50% and 20 ng g^−1^ in the upper soil layer are depicted in Figure 5c. The residues of metalaxyl and zoxamide decreased to a greater extent than those of boscalid, dimethomorph and azolic compounds from 2022 to 2023. Therefore, the dissipation of total residues at a given sampling site also depends on the particular compounds existing in each of the considered vineyards.

### 3.3. Soil Microbiome

The microbial diversity in vineyard soils is influenced by several factors including geography, climate or plant–microbe interactions, as well as anthropogenic factors such as farming practices, which are an important driver of bacterial and fungal communities [5]. In addition, soil biodiversity is linked to ecosystem stability. Thus, high genetic variability enables resistance to environmental changes [73]. Practices such as fertilization, different agricultural management systems or the application of fungicides can cause a loss of species with key functions in the ecosystem.

#### 3.3.1. Transition from Conventional to Organic Management Improves Soil Microbiome Indicators

**Figure 6:**
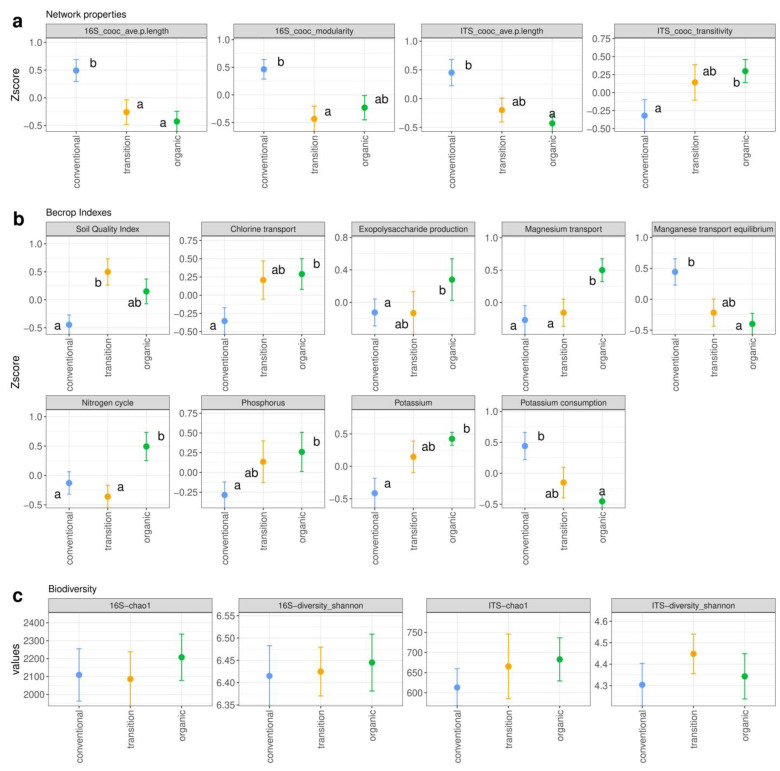
Network properties (**a**), microbiome BeCrop^®^ indexes (**b**) and alpha diversity metrics (**c**) factorial plots depending on the management practice. Superscript letters indicate statistically different groups (adj. *p*-value < 0.05). Results are shown in terms of mean and model standard error (bars).

The study of a microbial network’s properties allows for an estimation of structural features of ecological interest to understand the microbiome’s functioning (i.e., niche specialization, the level of competition and functional redundancy) [51]. Generally, conventional practices lead to highly clustered and specialized networks. These networks may be more susceptible to perturbation since the loss of a species specialized in a unique ecosystem process results in the loss of that function [74]. In contrast, organic practices have been related to more complex networks with a higher level of connectivity and abundance of keystone taxa [75]. All our results for network properties in the current study matched our expectations (Figure 6a). First, bacterial networks showed a significantly lower co-occurrence modularity and average path length in transitional and ecological management systems than the conventional system. This highlights a highly interconnected and cooperative network. Fungal networks had a lower average path length and higher level of transitivity in the transitional and ecological management systems. These results outlined the same mechanisms as described for prokaryotes. Our findings confirm that sustainable management systems in vineyard soils promote more collaborative microbial networks. Hence, these networks could be related to a higher level of resistance and resilience to different stress conditions, as reported by Ortiz-Álvarez et al. [51]. The network properties for bacterial communities were also significantly affected by the depth level, with a lower co-occurrence modularity and average path length in the top soil fraction (Figure S4a).

The BeCrop^®^ indexes provide comprehensive information regarding key features of soil health from different perspectives. These indexes include carbon, nitrogen, potassium and phosphorous as well as other micronutrient metabolism indexes along with hormone, stress and biocontrol indicators. Our results indicated that the BeCrop^®^ Soil Quality Index (SQI) reflected agronomic practices, yielding a lower SQI score for conventional practices and a higher score for transitional/organic management systems (Figure 6b). Regarding particular nutrients, organic management significantly improved nitrogen cycling and overall potassium and phosphorous metabolism when compared to conventional management (Figure 6b). Organic management also improved some micronutrient transport, such as magnesium and chlorine transport, while enhancing exopolysaccharide production (Figure 6b). The transitional system had intermediate values for some indexes (chlorine transport, exopolysaccharide production and potassium-related indexes). Conversely, it had the same magnitude as conventional management for other indexes (nitrogen cycle and magnesium transport) (Figure 6b). Soil depth also had an impact on several of the BeCrop^®^ indexes. For instance, fermentation, iron assimilation, magnesium transport, methanogenesis, nitrogen cycling, siderophore production and the sulphur cycle equilibrium were promoted in the top soils. In contrast, inorganic P and potassium solubilization was promoted at the 20 cm depth (Figure S4b).

The BeCrop^®^ indicators contrasted with some of our physicochemical results. Thus, the contents of N and C were higher in the upper soil layer, but there were no significant differences between management systems (Table 1). However, the nitrogen cycle could be improved in the organic vineyards via microbial communities. Similarly, phosphorus was favored under sustainable management even though no differences were found for the P content between the management systems or between the depths. However, regarding the SB, the physicochemical results showed the higher availability of exchangeable Ca, Mg and K in organic soils (Figure 2). These results agree with the BeCrop^®^ indexes related to higher values of magnesium transport and potassium, as well as lower potassium consumption in organic samples (Figure 6b).

The role of organic farming to ensure biodiversity and sustainability in viniculture is a debate of great interest. Our results evidenced the positive influence of organic management on soil acidity and the availability of Ca, Mg and Zn. Moreover, organic management showed more compact microbial networks and improved certain soil microbiome quality indicators (nitrogen cycling, overall potassium and phosphorous metabolism, magnesium and chlorine transport and exopolysaccharide production, as shown by the BeCrop^®^ indicators). In this sense, other authors have also concluded that organic farming promotes biodiversity and natural pest control. However, organic management was also associated with lower production levels. Hence, it needs to be supplemented by management options to balance biodiversity conservation and the simultaneous provision of multiple ecosystem services [76].

#### 3.3.2. Factors Affecting Microbial Diversity

The results of the alpha diversity in the vineyards’ soils from DOP Ribeiro are shown in Figure 6c. All of the vineyard soils harbored a higher diversity in terms of the Richness Chao 1 index and Shannon index (H) of prokaryotes (Chao1 = 2011 and H = 6.38, respectively) compared to fungal communities (Chao1 = 649 and H = 4.35, respectively). The values were lower than those reported at a global scale, including those for Spain [9]. However, they were similar to those found in Spain at the local scale [77] and higher than the fungal indexes reported in bulk soils from La Rioja [78]. Notably, there were no significant differences between management practices, although a tendency to a higher level of diversity could be observed in organic management systems for prokaryotes in both indexes. Similarly, the Chao 1 index for fungal populations was higher in the vineyards with organic management. However, the Shannon index (which considers the number and the abundance of species) was higher in the transitional vineyards (Figure 6c). Similarly, the available data on the influence of management systems on microbial populations indicated no differences or contradictory results. For instance, higher richness values and Shannon index scores for soil bacterial communities in conventional and/or biodynamic vineyards compared to organic vineyards have been observed [17,72]. Similarly, the opposite has been observed for fungal communities, with no significant differences between management systems [72]. Higher contents of C and N were related to a higher level of bacterial diversity whereas pH levels did not have an effect [5]. Accordingly, in our study, organic soils had a higher pH, but no significant differences were observed for either the C and N contents or the microbial diversity. The ratio of C:N was the main factor explaining microbiome differences between vineyards from regions in Argentina [79]. However, the ratio of C:N as well as the available P content (Pbray) did not differ among soil sample depths and management practices in vineyard soils from DOP Ribeiro (Table 1). Regarding fungal composition, significant differences were reported for vineyard soils under organic, biodynamic, and conventional or integrated practices, but not for the number of species [22,80]. The latter described a decrease in the level of bacteria richness in integrated vineyards compared to organic ones but similar compositions.

**Figure 7:**
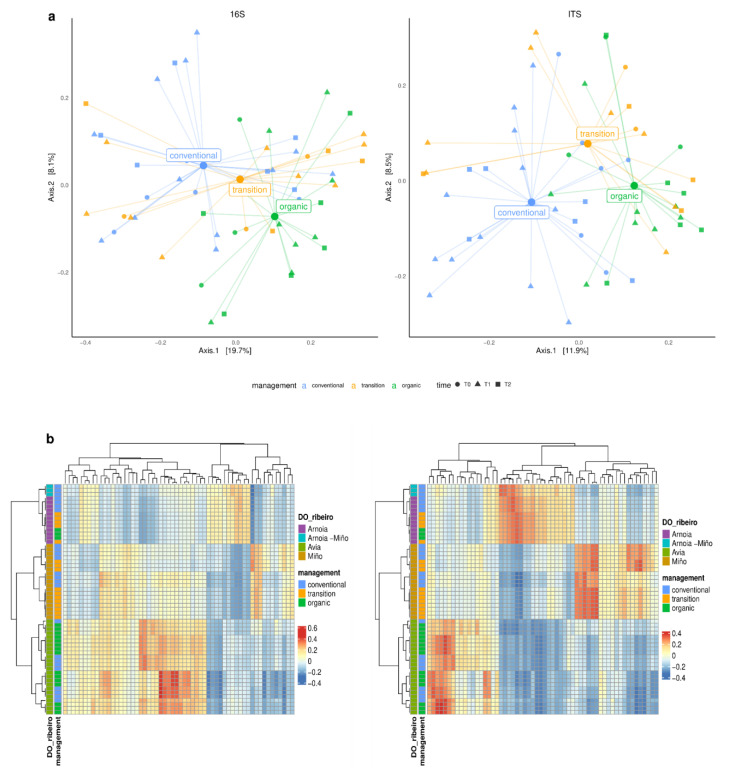
Principal coordinate analysis (PCoA) of the microbial community based on Bray–Curtis distances for 16S and ITS markers annotated by management and time point. Management labels are placed on their corresponding centroids (**a**). Clustering correlation of geographical and beta diversity distances for 16S and ITS markers, respectively. Each row/column is a sample (**b**).

Regarding beta diversity, our PCoA showed management-dependent clusters for both prokaryote and fungal communities (Figure 7a). This is in contrast to the similarity in fungal compositions between organic and conventional vineyards from Spain [51], although biodynamic vineyards differed. Other authors have reported that pH and the C:N ratio were the factors that most strongly correlated with the microbial beta diversity of bacteria in five vineyards from the USA [14].

When correlating microbiome composition patterns to effective geographical distances, despite different agricultural practices, the microbial terroir within DOP Ribeiro could be clearly defined (Figure 7b). The Arnoia and Anoia–Miño clusters highly differ from those of Miño and Avia. A spatial distribution of the soil microbiome has been reported at different scales, including across continents [9], countries and regions [5,6,7,79], as well as between and even within vineyards [4,8]. Moreover, it is known that the vineyard soil determines the microorganisms present in grapes, and the grapevine microbiome influences the fermentative process; therefore, it is related to regional wine characteristics [10,11,12,14].

Previous studies carried out in Evega have shown the existence of biogeographic patterns in cultivable yeasts in grapes and musts from different DOPs in Galicia [31,32]. In this work, we have used next-generation sequencing (NGS) and bioinformatics approaches to evidence, for the first time, differences in the microbial community for the Miño, Avia and Arnoia subzones within DOP Ribeiro. These microbial terroirs could explain the complex wine diversity of this region. Furthermore, these findings could contribute to recognize different subzones in DOP Ribeiro as it happens in other DOP in Galicia like DOP Rías Baixas or DOP Ribeira Sacra.

Although geographical location is one of the main drivers of bacterial and fungal microbial communities in soils, season (sampling time) and agricultural management as well as soil properties also have an effect [5,17]. The influence of the zone, management, time and depth on the microbiota in vineyard soils from DOP Ribeiro is summarized in Table 3. Moreover, our PERMANOVA analysis confirmed the effect of all of these factors, with area being the more relevant factor for bacteria whereas time was the main driver for fungal beta diversity (Table 3). Management also had a significant effect on microbial communities, whereas depth was the factor with the weakest impact. In addition, soil depth had a greater influence on bacterial communities than on fungal communities, as previously reported by Wright et al. [81]. The effect of the sampling time (summer or winter) on fungal populations could be related to the differences in the content of pesticide residues, mainly fungicides, in soil samples (Figure 5b). In fact, a study including soils samples from vineyards during different seasons was carried out in an area with low-impact fungicide treatments and did not find differences in terms of fungal diversity [77].

#### 3.3.3. Preserved and Variable Taxonomic Fractions across DOP Ribeiro with Different Management Practices

**Figure 8:**
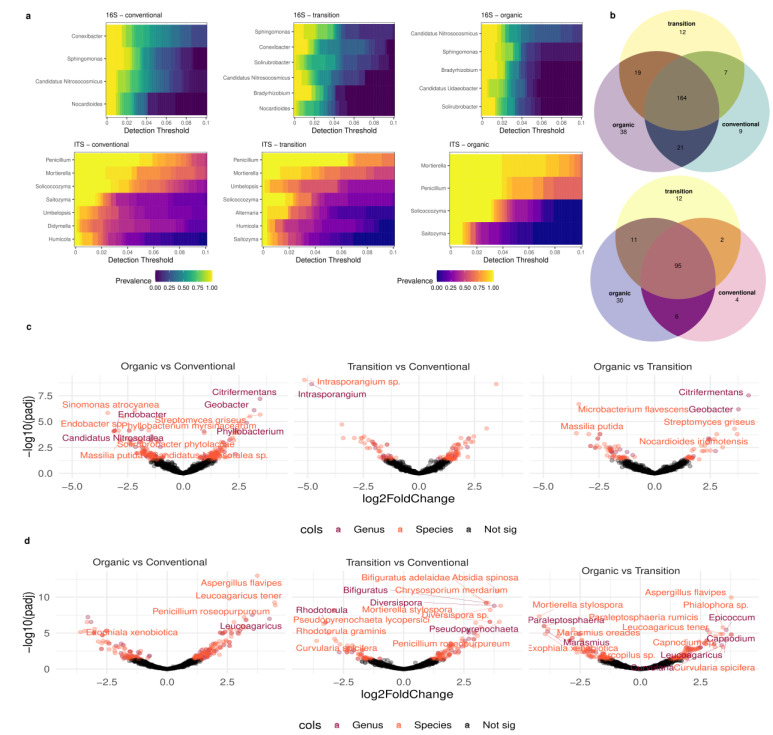
Heatmaps showing the genus prevalence proportion across different detection thresholds in ecologic, transitional and conventional management systems for 16S and ITS markers (**a**). Number of shared and exclusive members of prokaryote (**top**) and fungal (**bottom**) taxa at genus level (**b**). Volcano plot showing differentially abundant taxa at different genus and species levels (adjusted *p*-value < 0.05) for 16S (**c**) and ITS (**d**). For contrast, results comparing all management systems are shown.

The results of the microbiome analysis in our samples from the vineyard soils under the different agricultural practices evidenced the relevant genera and species for each management system. Figure 8a shows the prevalence of the most relevant genera/species under organic, transitional and conventional management for the 16S and ITS markers. The 16S marker included the archaea genus *Nitrosocosmicus*, which is the most robust core member in ecologic management while occupying a less prevalent position in transitional and conventional management. This genus has been reported to be a plant-growth-promoting archaeon by oxidizing N into plant-bioavailable forms and is a kind of ammonia-oxidizing archaeon (AOA) [82]. Hence, we hypothesize that organic management promotes overall nitrogen-cycle-related taxa, as highlighted by the Nitrogen Cycle BeCrop^®^ index score increases mentioned above (Figure 6b). *Sphingomonas* was the most relevant genus in the transitional samples and the second in the other two management systems. In addition, *Bradyrhizobium* is a core member, not present in the conventional system, but emerging in the transitional system and increasing in prevalence in the organic management system (Figure 8a). *Udaeobacter* is relevant in organic management whereas *Solirubrobacter* was prevalent in organic and transitional management but not in conventional management. Conversely, *Conexibacter* and *Nocardioides* appeared as core members in transitional and conventional management, but they were not as important in organic management. Our results agreed partially with those for vineyards in Chile, in which *Conexibacter* and *Bradyrhizobium*, among others, were the most abundant genera [83].

Regarding the fungi core microbiome, *Mortierella* was the most robust core member in the ecological management system, while *Penicillium* dominated the transitional and conventional management systems (Figure 8a). *Solicoccozyma* and *Saitozyma* were present in all of the management systems, whereas *Humicola* was characteristic of the conventional and transitional management systems, but not the organic vineyards. *Alternaria* was found to be a core member in transitional management systems while *Didimella* in was a core member in conventional management systems. Notably, *Mortierella* was found to be differentially abundant in the ecological and transitional management systems when compared to the conventional management system (Table 4). Several positive functions of *Mortierella* strains have been described in soils (for a review, see [84]). *Mortierella* are plant-growth-promoting fungi (PGPF) that improve a plant’s access to bioavailable forms of P and Fe, positively interact with arbuscular mycorrhizal fungi (AMF), promote the synthesis of phytohormones and offer plant protection against pathogens and adverse conditions. Moreover, they increase nutrient uptake efficiency, significantly enhancing crop yield. These ecological services could explain the differences found in some of the BeCrop^®^ indexes for the organic and/or transitional management systems.

Figure 8b represents the number of shared and exclusive members of prokaryote (top) and fungal (bottom) taxa at the genus level. Vineyards with ecological management had the highest number of exclusive genera both for prokaryote (38 vs. 12 and 9) and fungal (30 vs. 14 and 4) communities when compared to the vineyards with transitional and conventional agricultural practices (Table S7).

Finally, volcano plots showing differentially abundant taxa at the genus and species level (adjusted *p*-value < 0.05) for 16S and ITS comparing all of the management systems are included in Figure 8c and 8d, respectively. *Streptomyces griseus* was highly differentially abundant in organic management when compared to both transitional and conventional management (Figure 8c and Table S8). *Streptomyces* are widely known as biocontrol and plant growth promoters, but this species has also been found to improve yield in grain crops [85]. Additionally, *Beauveria* and *Metarhizum* were found to be differentially abundant in the ecologic and transitional management systems when compared to the conventional management system (Table S8). Both genera are known for their entomopathogenic effect and are used in biocontrol products against different insect infestations [86].

Noteworthy, *Diversispora*, an arbuscular mycorrhizal taxon, was found to be differentially abundant in the transitional management system (Figure 8d). Arbuscular mycorrhizal fungi live in endosymbiosis with many vascular plants, providing the plant with nutrients in exchange for carbon [87].

## 4. Final Remarks and Conclusions

The use of more sustainable agricultural practices (organic management) in DOP Ribeiro vineyards positively influences soil conditions. Thus, a trend of decreasing soil acidity and an increase in exchangeable basic cations like Ca and Mg and micronutrients like Zn is observed in organic vineyards when compared with conventional ones. Although these factors are important drivers of soil microbial diversity, there were no significant differences in alpha diversity between the management systems. However, the sustainable practices led to more collaborative microbial networks, which may enhance plant resistance and resilience to different stress conditions. In addition, the soil nutrient availability (N cycling, P and K metabolism, Mg and chlorine transport) and soil quality index were higher in the vineyards with organic management systems.

The presence of pesticide residues in the organic vineyards was anecdotal, as expected. For the conventional and transitional vineyards, a reduction in residue concentration from summer to winter was observed in most cases. However, a reduction in pesticides in the soils from the vineyards transitioning from conventional to organic management was not so clear. This may be due to the long persistence in soils of some residues and highlights the fact that longer periods of conversion are necessary to ensure the absence of residues in vineyard soils. Therefore, further research is required by increasing both the number of soils examined for each management system and the duration of organic management practices to confirm their impact on the soil condition, residue content and microbial diversity.

Regarding beta diversity, the type of management explained the variations in both prokaryote and fungal communities. Nevertheless, the main factor for microbial differentiation was the geographical distance for prokaryotes and the time of sampling (summer or winter) for fungi. Moreover, the analysis of microbiome evidenced differential core genera and species for each management system with organic soils having the highest number of exclusive genera.

Finally, the correlation of the microbiome to the different subzones within DOP Ribeiro evidenced the existence of a *microbial terroir* associated with the valleys of the Avia, Arnoia and Miño rivers, which could explain the complex and unique wine diversity of this region.

## Figures and Tables

**Table 1:** Soil reaction and organic indicators of soils according to depth and management practices.

	Soil Depth	0–5 cm			5–20 cm		
	Management	Conventional	Transitional	Organic	Conventional	Transitional	Organic
pHw	avg ± sd	5.6 ± 0.4 ^b^	5.8 ± 0.6 ^ab^	6.0 ± 0.5 ^a^	5.4 ± 0.4 ^b^	5.8 ± 0.5 ^ab^	6.0 ± 0.4 ^a^
	max − min	6.7 − 5.0	6.6 − 4.9	6.9 − 5.2	6.0 − 4.8	6.5 − 5.0	6.6 − 5.3
pHk	avg ± sd	4.9 ± 0.3 ^a^	5.2 ± 0.4 ^a^	5.1 ± 0.4 ^a^	4.7 ± 0.3 ^b^	5.1 ± 0.5 ^a^	5.1 ± 0.3 ^a^
	max − min	5.5 − 4.4	5.7 − 4.4	5.7 − 4.5	5.3 − 4.2	5.6 − 4.0	5.6 − 4.6
C (g kg^−1^)	avg ± sd	21.1 ± 6.8 ^a^	26.7 ± 17.3 ^a^	24.6 ± 5.7 ^a^	14.1 ± 5.7 ^a^	13.4 ± 5.8 ^a^	12.7 ± 1.6 ^a^
	max − min	32.5 − 11.9	58.6 − 11.5	38.7 − 17.9	23.3 − 5.4	22.1 − 7.0	15.3 − 10.5
N (g kg^−1^)	avg ± sd	1.6 ± 0.6 ^a^	2.2 ± 1.5 ^a^	2.0 ± 0.5 ^a^	1.1 ± 0.5 ^a^	1.0 ± 0.4 ^a^	1.0 ± 0.2 ^a^
	max − min	2.4 − 0.8	5.0 − 0.8	3.0 − 1.4	1.8 − 0.4	1.7 − 0.5	1.3 − 0.8
C/N	avg ± sd	13.4 ± 1.2 ^a^	12.5 ± 1.0 ^a^	12.6 ± 1.0 ^a^	13.5 ± 1.6 ^a^	13.0 ± 1.0 ^a^	13.4 ± 1.3 ^a^
	max − min	16.0 − 11.7	14.0 − 11.5	14.2 − 10.9	16.1 − 11.6	14.8 − 12.1	16.3 − 11.6
P (mg kg^−1^)	avg ± sd	46.3 ± 35.1 ^a^	44.0 ± 25.6 ^a^	39.1 ± 16.1 ^a^	45.3 ± 22.5 ^a^	51.8 ± 44.3 ^a^	34.3 ± 15.7 ^a^
	max − min	144.6 − 15.5	101.8 − 18.4	75.7 − 21.5	81.2 − 23.1	149.1 − 14.2	61.1 − 18.0

For each soil property and depth sample, different letters show statistical differences (*p* < 0.05) according to Mann–Whitney (U) test.

**Table 2:** Total contents of Fe, Mn, Cu and Zn according to depth and management practices.

	Soil Depth	0–5 cm			5–20 cm		
	Management	Conventional	Transitional	Organic	Conventional	Transitional	Organic
Fe_T_ (g kg^−1^)	avg ± sd	28.7 ± 10.6 ^a^	31.8 ± 8.2 ^a^	27.6 ± 7.0 ^a^	30.5 ± 11.54 ^a^	31.5 ± 9.5 ^a^	29.60 ± 6.0 ^a^
	max − min	49.5 − 19.7	43.4 − 24.6	35.9 − 19.1	52.9 − 19.7	45.1 − 23.3	37.6 − 23.7
Mn_T_ (mg kg^−1^)	avg ± sd	233.5 ± 132.1 ^a^	253.0 ± 50.4 ^a^	426.1 ± 146.4 ^a^	218.3 ± 130 ^b^	233.8 ± 89.5 ^ab^	483.1 ± 119.8 ^a^
	max − min	458.9 − 83.7	322.5 − 213.0	595.6 − 247.2	431.2 − 69.1	361.5 − 166.8	556.8 − 274.0
Cu_T_ (mg kg^−1^)	avg ± sd	176.0 ± 96.9 ^a^	217.0 ± 163.3 ^a^	167.2 ± 58.3 ^a^	172.1 ± 113.7 ^a^	165.1 ± 154.1 ^a^	132.7 ± 57.4 ^a^
	max − min	395.3 − 69.1	571.4 − 54.1	240.5 − 91.2	397.7 − 58.0	473.2 − 24.5	206.7 − 50.3
Zn_T_ (mg kg^−1^)	avg ± sd	71.1 ± 18.9 ^b^	127.8 ± 77.1 ^a^	93.4 ± 15.4 ^a^	63.4 ± 18.5 ^a^	76.3 ± 11.4 ^a^	79.4 ± 12.0 ^a^
	max − min	105.4 − 43.8	287.5 − 73.3	127.5 − 79.4	89.0 − 37.0	91.7 − 60.5	106.3 − 66.8

For each total content and depth sample, different letters show statistical differences (*p* < 0.05) according to Mann–Whitney (U) test.

**Table 3:** PERMANOVA analysis on microbiome data composition for 16S and ITS markers considering DOP Ribeiro zone, management, depth and time as factors.

Marker	Factor	*p*-Value	R^2^ (%)
16S	Ribeiro zone	0.01	10.71
	management	0.01	7.54
	time	0.01	8.96
	depth	0.01	5.84
ITS	Ribeiro zone	0.01	8.78
	management	0.01	8.41
	time	0.01	10.25
	depth	0.02	2.60

**Table 4:** Highly differentially abundant *Mortierella* spp. in organic and transitional management systems when compared to conventional systems and a comparison between them. The log2Fold change is greater than 2 (absolute value) and adjusted *p*-value is <0.05. A positive fold change indicates that a taxon was differentially abundant in the organic or transitional treatment compared to the conventional treatment, or in organic treatment compared to transitional treatment; a negative fold change indicates a taxon was differentially abundant in the transitional management system compared to organic system or in conventional system compared to organic or transitional systems.

Mortierella spp.	Management Comparison	Fold Change (log)	adj-*p*-Value
*Mortierella amoeboidea*	organic_vs._conventional	2.17	1.02 × 10^−2^
*Mortierella globulifera*	organic_vs._conventional	2.12	3.15 × 10^−4^
*Mortierella sclerotiella*	transition_vs._conventional	2.98	3.79 × 10^−7^
*Mortierella stylospora*	transition_vs._conventional	4.02	1.60 × 10^−9^
*Mortierella amoeboidea*	organic_vs._transition	3.10	8.80 × 10^−4^
*Mortierella fatshederae*	organic_vs._transition	−2.01	1.58 × 10^−2^
*Mortierella gamsii*	organic _vs._transition	−2.12	1.58 × 10^−2^
*Mortierella sclerotiella*	organic _vs._transition	−2.10	9.68 × 10^−4^
*Mortierella stylospora*	organic _vs._transition	−4.20	4.79 × 10^−8^

## Data Availability

The data presented in this study are available on request from the corresponding author due to privacy.

## Supplementary Materials

The following supporting information can be downloaded at: https://www.mdpi.com/article/10.3390/microorganisms12030595/s1, Table S1: Mean temperature at 1.5 m above grand registered by the three stations of the study area provided from meteogalicia.gal; Table S2: Total rain (L m^−2^) registered in the three stations of the study area provided from meteogalicia.gal; Table S3: Retention times and MRM conditions of native pesticides and selected surrogate standards; Table S4: Global recoveries of the analytical procedure in two different vineyard soils spiked at two concentration levels referred to freeze-dried soil. Data for samples spiked at 50 ng g^−1^ (n = 6 replicates); Table S5: BeCrop^®^ indexes list and definition grouped by their respective category; Table S6: Summary of concentrations for pesticides in transition and conventional managed vineyards. Table S7: Complete list of shared taxa of bacteria (16S) and fungal (ITS) taxa at genus level; Table S8: Complete list of differentially abundant taxa in organic and transition management when compared to conventional and compared between them; Figure S1: Fe and Al fractionation; Figure S2: Released concentrations of Cu after fractionation study; Figure S3: Distribution of total Cu and Zn among soil different fractions; Figure S4: Network properties (panel a) and microbiome BeCrop^®^ indexes (panel b) factorial plots depending on depth level. Superscript letters indicate statistically different groups (adj. *p*-value < 0.05).
