# Supplementary material for: Physicochemical Properties and Microbiome of Vineyard Soils from DOP Ribeiro (NW Spain) Are Influenced by Agricultural Management
Source: Microorganisms. 2024 Mar 16;12(3):595. doi: 10.3390/microorganisms12030595 (PMC10974217; doi:10.3390/microorganisms12030595)
Supplement: Supplementary file 1 [file microorganisms-12-00595-s001.zip › Figure S4 Network properties.pdf]

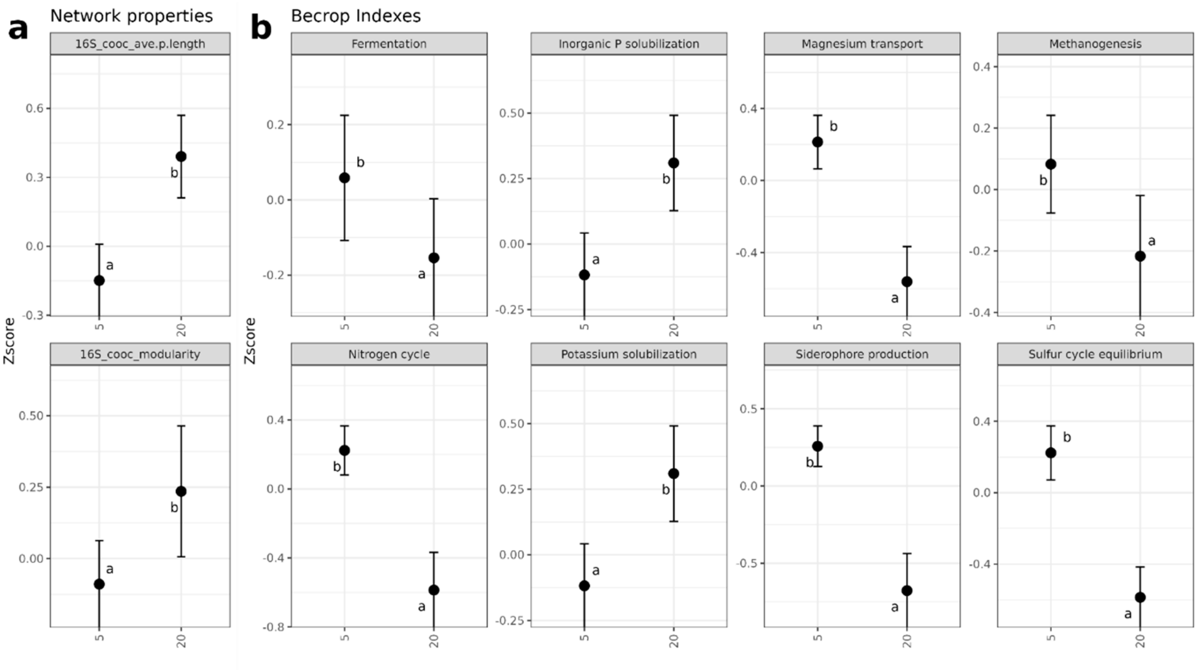

Figure S4 Network properties (panel a) and microbiome BeCrop® indexes (panel b) factorial plots depending on depth level. Superscript letters indicate statistically different groups (adj. p-value < 0.05).
