# Supplementary material for: Physicochemical Properties and Microbiome of Vineyard Soils from DOP Ribeiro (NW Spain) Are Influenced by Agricultural Management
Source: Microorganisms. 2024 Mar 16;12(3):595. doi: 10.3390/microorganisms12030595 (PMC10974217; doi:10.3390/microorganisms12030595)

Table S1. Mean temperature at 1.5m above grand registered by the three stations of the study area provided from meteogalicia.gal (Station 1: EVEGA Leiro, Station 2: Remuíño Arnoia and Station 3: Prado-Castrelo de Miño).

			Mean temperature at 1.5m above ground (°C)		
YEAR	SEASON	MONTH	Station 1	Station 2	Station 3
2022	WINTER	JANUARY	5.3	5	6.1
		FEBRUARY	8	7.9	8.6
		MARCH	10.4	10.3	10.8
	SPRING	APRIL	12.2	12.3	12.6
		MAY	18.1	17.9	18.4
		JUNE	19	18.8	19.1
	SUMMER	JULY	24.3	24.1	24.6
		AUGUST	23.8	23.6	24
		SEPTEMBER	19.3	18.8	19.5
	AUTUMN	OCTOBER	16.5	16.4	17
		NOVEMBER	11.5	11.4	11.9
		DECEMBER	10.8	10.9	11.4
2023	WINTER	JANUARY	7.1	7	7.6
		FEBRUARY	6.6	6.3	7.2
		MARCH	11.8	11.9	12.2

Table S2. Total rain (L m^{-2}) registered in the three stations of the study area provided from meteogalicia.gal (Station 1: EVEGA Leiro, Station 2: Remuíño Arnoia and Station 3: Prado-Castrelo de Miño).

			Total rain (L m^{-2})		
YEAR	SEASON	MONTH	Station 1	Station 2	Station 3
2022	WINTER	JANUARY	15.4	22.6	16.8
		FEBRUARY	23.6	30	14.6
		MARCH	85.2	91.6	87.1
	SPRING	APRIL	79.6	76.4	65.1
		MAY	14.4	14.4	13.5
		JUNE	69	96.8	54
	SUMMER	JULY	9.6	1.4	9.3
		AUGUST	7	2.8	14.6
		SEPTEMBER	56.8	73.8	57.2
	AUTUMN	OCTOBER	298	248	243.6
		NOVEMBER	190	242.2	217.5
		DECEMBER	308	275.3	235.3
2023	WINTER	JANUARY	191.6	226.8	199.5
		FEBRUARY	15	19	16.7
		MARCH	80.2	120.4	66.1

Figure S1. Fe and Al fractionation

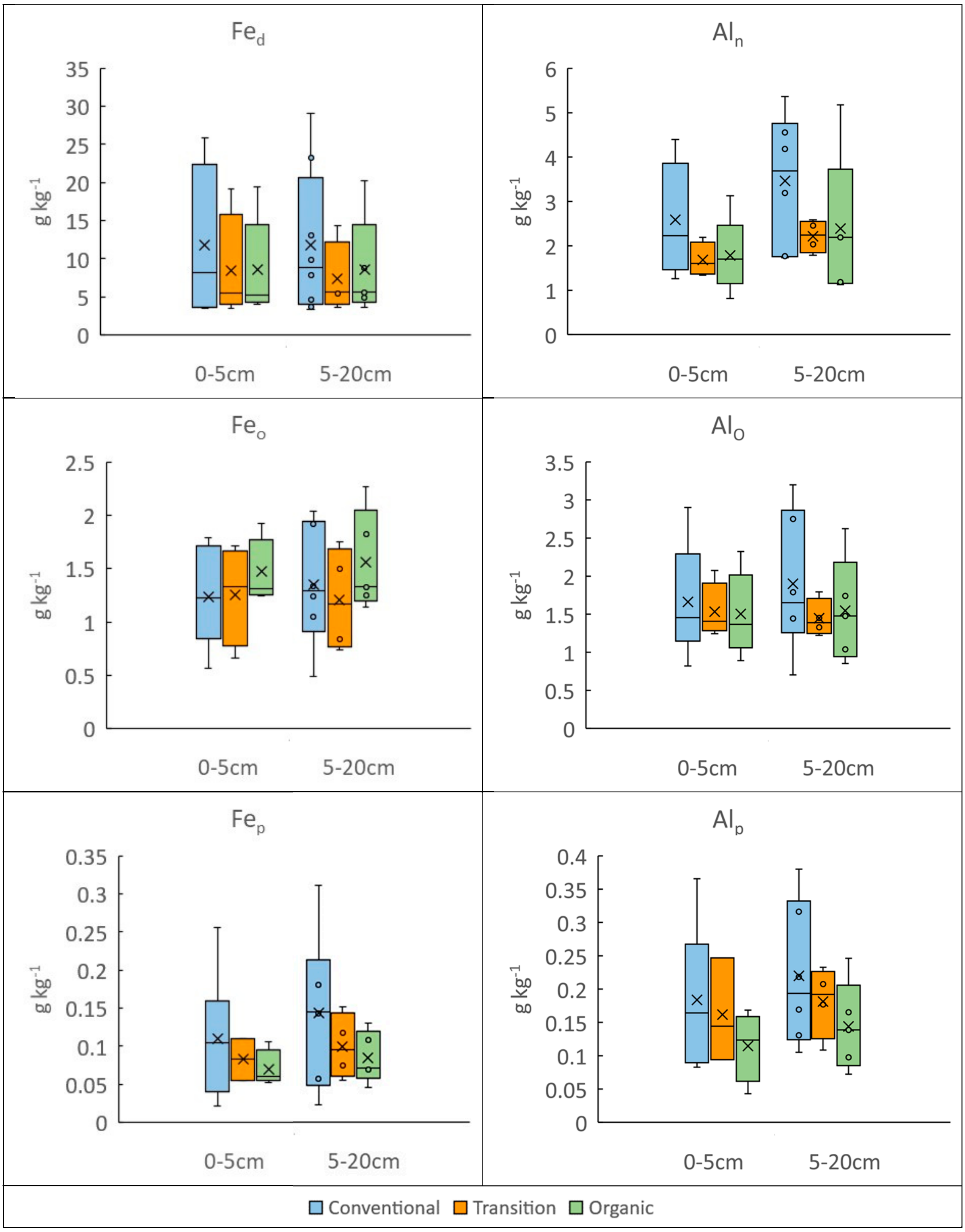

Figure S2. Released concentrations of Cu after fractionation study

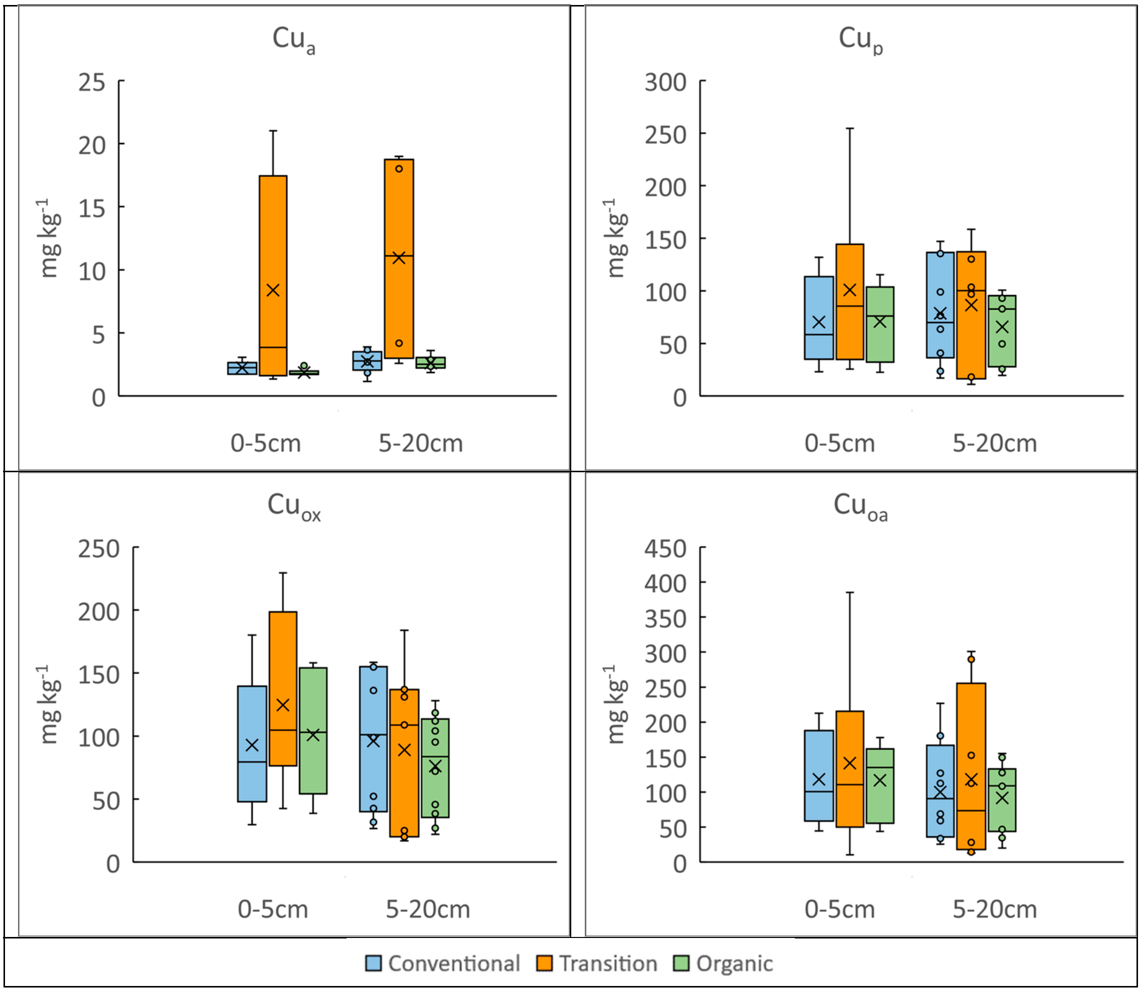

Figure S3. Distribution of total Cu and Zn among soil different fractions

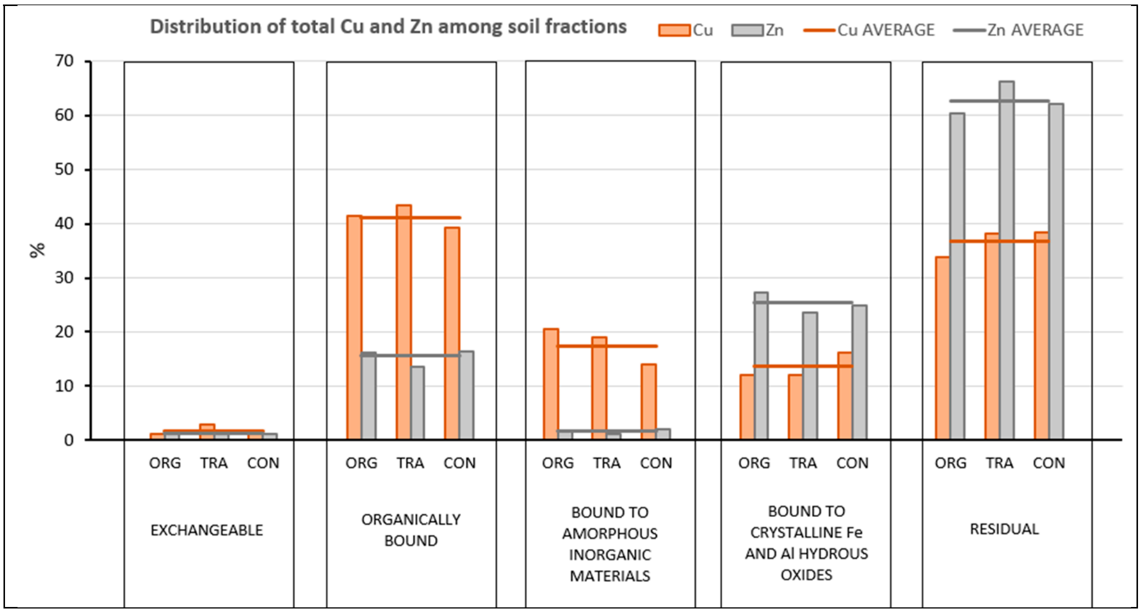

Supplement: Supplementary file 1 [file microorganisms-12-00595-s001.zip › Supplementary material-FQ soils.pdf]
