# Supplementary material for: Physicochemical Properties and Microbiome of Vineyard Soils from DOP Ribeiro (NW Spain) Are Influenced by Agricultural Management
Source: Microorganisms. 2024 Mar 16;12(3):595. doi: 10.3390/microorganisms12030595 (PMC10974217; doi:10.3390/microorganisms12030595)
Supplement: Supplementary file 1 [file microorganisms-12-00595-s001.zip › Table S5-BeCrop Indexes.pdf]

Table S5. BeCrop® indexes list and definition grouped by their respective category.

**BeCrop® index category**	**BeCrop® index**	**Description**
Soil Quality	Biodiversity	Alpha diversity for both microbial communities
	Biodiversity (bacteria)	Alpha diversity for bacteria communities
	Biodiversity (fungi)	Alpha diversity for fungi communities
	Functional diversity	Amount of different ecological roles performed in soil by the microbial community
	Resilience	Measure of cluster structure of the active community, with dense and unclustered networks (high resistance score) indicating a broad ability to respond to environmental challenges
	Soil Quality Index	Bioindicator based on soil microbiome ecology that changes based on agriculture management intensification
Hormones Impact	Auxin production (IAA)	Responsible for cell division and elongation
	Cytokinin production (CK)	Responsible for cell proliferation and differentiation
	Gibberellin production (GA)	Responsible for elongation, germination and flowering
Stress Impact	Abscisic acid (ABA)	Growth regulation, Plant resistance
	ACC deaminase (ACC-d)	Pathogen protection. Drought protection
	Exopolysaccharide production	Nutrient trap, Salinity protection
	Heavy metal solubilization	Bioremediation, Detoxification
	Salicylic acid (SA)	Alleviate water stress, Salinity protection
	Salt tolerance	Alleviate water stress, Root Growth
	Siderophore production	Iron nutrition, Biofertilization
Biocontrol Impact	Bactericide agents	Microbial species capable of preventing pathogenic species from taking hold or proliferating
	Fungicide agents	
	Insecticide agents	
	Nematicide agents	
Nitrogen Metabolism	Nitrogen	Summary of nitrogen indexes. Nitrogen plays a fundamental role in crop yield
	Inorganic nitrogen consumption	Uptake of nitrogen by plants and microbes

	Inorganic nitrogen release	Process of mineralization, microbial transformation of organic Nitrogen compounds to inorganic that serve as plant nutrients
	Nitrogen cycle	Steps of nitrogen cycles: ammonification, nitrification, denitrification and anaerobic ammonia oxidation
Phosphorus Metabolism	Phosphorus	Summary of Phosphorus indexes. Nutrient required for the regulation of protein synthesis and plant growth
	Inorganic P consumption	Uptake of Phosphorus by plants and microbes
	Inorganic P solubilization	Microbes can convert insoluble phosphorus in the soil into available forms that plant can absorb
	Organic P assimilation	Intermediate step of phosphorus cycle to pass from organic to inorganic forms
Potassium Metabolism	Potassium	Summary of potassium indexes. Potassium is involved in the regulation of several microbial activities, such as the production of sugars and proteins involved in crop evapotranspiration.
	Potassium consumption	Potassium uptake by plants and microbes
	Potassium solubilization	Process of dissolving insoluble potassium in the soil into available forms for plants
Carbon Metabolism	Carbon	Summary of carbon indexes. Carbon play an important role in soil fertility
	Aerobic respiration	Process in which microbes use organic compounds in oxygenated conditions, releasing CO_2
	Carbon fixation	what microbes need to grow
	Fermentation	Process in which microbes gain energy from organic compounds in non-oxygenated conditions, releasing CO_2
	Organic matter release	Process in which soil microorganisms decompose vegetal debris, releasing diverse mineral nutrients.
	Methanogenesis	The formation of Methane (CH_4) by microbes
Micronutrient Metabolism	Calcium transport	Contributes to soil fertility
	Chlorine transport	Important micronutrient that takes part in processes such as disease resistance and tolerance
	Copper export	Potential toxic micronutrient
	Iron assimilation	Crucial for enzymatic activities such as nitrogen fixation
	Magnesium transport	Nutrient involved in enzyme activities and structural stabilization of tissue
	Manganese transport equilibrium	Involved in photosynthesis respiration and nitrogen assimilation

	Sulfur cycle equilibrium	Essential nutrient to maintain good health and high yields
	Zinc transport equilibrium	Zinc transport equilibrium. Crucial for plant development
Diseases	*All major and minor diseases*	Level of risk of specific crop pathogens
